# Chidamide plus venetoclax as effective and safe maintenance therapy in high-risk acute myeloid leukemia and myelodysplastic syndrome post-transplantation

**DOI:** 10.1007/s00277-026-07221-8

**Published:** 2026-08-07

**Authors:** Cuicui Lyu, Haotian Meng, Yedi Pu, Xianshuang Luan, Hairong Lyu, Xia Xiao, Mingfeng Zhao

**Affiliations:** Department of Hematology, Tianjin First Central Hospital, Tianjin Thrombosis and Hemostasis Institute, No.2 Baoshan West Road, Xiqing District, Tianjin, 300192 China

**Keywords:** Acute myeloid leukemia, Myelodysplastic syndromes, Chidamide, Venetoclax, Maintenance therapy, Hematopoietic stem cell transplantation

## Abstract

Relapse after allogeneic hematopoietic stem cell transplantation (allo-HSCT) remains the leading cause of treatment failure in high-risk acute myeloid leukemia (AML) and myelodysplastic syndrome (MDS), yet effective maintenance strategies are currently lacking. The combination of chidamide (CHI) and venetoclax (VEN) represents a novel approach. However, real-world data on its use are limited. In this study, eighteen high-risk transplant recipients received maintenance therapy with CHI (5 mg/day on days 1–7 or 1–14) plus VEN (100 mg on day 1, then 200 mg on days 2–7 or 2–14) every 2 months. Endpoints included overall survival (OS), disease-free survival (DFS), cumulative incidence of relapse (CIR), non-relapse mortality (NRM), and safety. After a median follow-up of 708 days, the 2-year OS and DFS were 73.9% and 56.6%, respectively. The 2-year CIR was 28.9%, and the 2-year NRM rate was 22.5%. The most common adverse events were hematologic, including neutropenia (13 episodes), thrombocytopenia (7), and anemia (7), all of which were manageable. No treatment-related mortality, tumor lysis syndrome, or severe acute graft-versus-host disease (GVHD) occurred. In conclusion, CHI plus VEN as post-transplant maintenance therapy shows encouraging efficacy and a manageable safety profile in high-risk AML/MDS patients, representing a promising strategy for relapse prevention.

## Introduction

 Allogeneic hematopoietic stem cell transplantation (allo-HSCT) serves as a potentially curative option for patients with high-risk acute myeloid leukemia (AML) and myelodysplastic syndromes (MDS) [1–3]. However, disease relapse remains the primary cause of treatment failure and mortality post-transplantation [4–6]. Patients who experience relapse face a dismal prognosis, with a median survival of only several months, posing a major challenge in current clinical practice [4–6]. Although maintenance strategies such as donor lymphocyte infusion and targeted therapies have been investigated to prevent relapse, their efficacy is often limited by an increased risk of graft-versus-host disease (GVHD), myelosuppressive toxicity, or inadequate response [5, 7]. Therefore, there is an urgent clinical need to develop novel maintenance therapies capable of effectively eliminating minimal residual disease (MRD) while maintaining a favorable safety and tolerability profile.

In recent years, significant progress in deciphering the pathological mechanisms of AML has accelerated the development of targeted and epigenetic therapies. Among these advances, the BCL-2 inhibitor venetoclax (VEN) has garnered considerable attention due to its potent anti-leukemic activity, which primarily functions by inducing apoptosis in tumor cells. As a result, VEN has emerged as a cornerstone agent in AML maintenance therapy, especially when used in combination with hypomethylating agents (Ven-HMAs) [8]. However, with the expanding clinical application of VEN-based therapies, the therapeutic efficacy of VEN is frequently compromised by primary resistance and clonal evolution. This limitation is particularly pronounced in certain AML subtypes, such as those exhibiting monocytic differentiation or specific genetic mutations, including alterations in *TP53*, *FLT3-ITD*, and *IDH*, which are consistently associated with suboptimal response rates and inferior clinical outcomes [9–13].

In contrast to VEN, the histone deacetylase inhibitor chidamide (CHI) acts by reversing epigenetic dysregulation and restoring the expression of tumor suppressor genes [14, 15]. CHI has demonstrated efficacy both as monotherapy and in combination with chemotherapy for relapsed/refractory AML(r/rAML) [16–18]. Notably, preclinical studies have revealed a marked synergistic effect between CHI and VEN in inducing apoptosis in AML cells [19, 20]. Mechanistically, CHI downregulates anti-apoptotic proteins such as MCL-1 and promotes DNA damage, thereby countering common resistance mechanisms observed with VEN [19, 20]. Moreover, this combination may enhance the graft-versus-leukemia (GVL) effect by modulating the immune microenvironment without increasing the risk of GVHD [19, 20]. Such mechanistic synergy provides a strong rationale for the combined use of CHI and VEN in the post-transplant maintenance setting. Nonetheless, clinical evidence regarding the efficacy, safety, and long-term outcomes of this combination after transplantation remains limited and warrants further validation through dedicated clinical studies.

In this study, we aim to systematically evaluate the real-world efficacy and safety of CHI combined with VEN as maintenance therapy following allo-HSCT in patients with high-risk AML/MDS, using a retrospective analysis. By analyzing clinical data from patients treated at our center, we seek to assess the efficacy of this combination in improving response rates and prolonging survival, while closely monitoring treatment-related toxicities and its impact on the risk of GVHD. This study is expected to provide valuable preliminary evidence supporting the use of this combination strategy in post-transplant maintenance therapy, thereby offering valuable insights for optimizing overall management and facilitating personalized treatment decisions in high-risk AML/MDS patients.

## Patients and methods

### Patients

This was a retrospective single center study conducted at the Department of Hematology, Tianjin First Central Hospital, covering the period from February 2021 to March 2025. Written informed consent was obtained from all eligible patients prior to allo-HSCT. During the pre-transplantation screening period, a total of 27 high risk AML/MDS patients were initially assessed for potential eligibility. Of these, 6 patients were excluded after transplantation due to severe complications that precluded maintenance therapy, and 3 patients declined to participate. After completion of the post-transplantation evaluation, 18 patients met all eligibility criteria for maintenance therapy and were formally enrolled into the final analytic cohort. Specific inclusion and exclusion criteria were defined according to the research objectives based on comprehensive treatment data.

Inclusion criteria for high-risk AML were as follows: (a) Age ≥ 60 years; (b) Prior history of MDS or myeloproliferative neoplasm (MPN); (c) Therapy-related AML or secondary AML; (d) White blood cell count ≥ 100 × 10⁹/L; (e) Central nervous system leukemia involvement; (f) Cytogenetic or molecular genetic classification into the poor-risk category, or presence of poor-prognosis gene mutations (e.g., *TP53*,RUNX1,ASXL1,BCOR, EZH2,SF3B1,SRSF2,STAG2,U2AF1,ZRSR2,FLT3-ITD with high-frequency allele ratio); (g) Failure to achieve complete remission after two cycles of standard induction chemotherapy; (h) Diagnosis of refractory or relapsed AML; (i) Positive MRD prior to transplantation. High-risk MDS was defined as a score ≥ 3 according to the World Health Organization Prognostic Scoring System (WPSS) or a score > 4.5 according to the Revised International Prognostic Scoring System (IPSS-R). Exclusion criteria were as follows: (a) severe concurrent infections; (b) severe GVHD; (c) disease relapse prior to the initiation of maintenance therapy; (d) any other condition deemed to make the patient unsuitable for maintenance therapy.

In addition to the diagnosis of high risk AML/MDS and successful allo-HSCT, patients were required to meet the following criteria for initiation of maintenance therapy: (a) adequate hematologic recovery between day + 30 and + 100 post-transplant, defined as absolute neutrophil count (ANC) ≥ 1.0 × 10⁹/L and platelet count (PLT) ≥ 50 × 10⁹/L without transfusion support; (b) no active uncontrolled infection or severe organ dysfunction (liver: ALT/AST ≤ 3 × ULN, total bilirubin ≤ 2 × ULN; renal: creatinine clearance ≥ 30 mL/min; cardiac: LVEF ≥ 50%); (c) Eastern Cooperative Oncology Group (ECOG) performance status ≤ 2; (d) absence of grade III to IV aGVHD or extensive chronic GVHD; (e) MRD status was assessed by multiparameter flow cytometry (sensitivity 0.01%) at day + 30 and prior to each treatment cycle. MRD negativity (< 0.01%) was considered to justify a prophylactic maintenance approach, whereas MRD positivity (≥ 0.01%) warranted a preemptive strategy. Exclusion criteria were as follows: (a) uncontrolled active infections, defined as persistent fever > 48 h or hemodynamic instability despite anti-infective therapy; (b) grade III to IV acute GVHD or extensive chronic GVHD requiring systemic immunosuppressive therapy; (c) disease relapse prior to the initiation of maintenance therapy; (d) severe organ dysfunction, including New York Heart Association class III or IV heart failure, Child-Pugh class B or C hepatic dysfunction, or estimated glomerular filtration rate < 30 mL/min/1.73 m²; (e) any other condition deemed by the investigator to make the patient unsuitable for maintenance therapy.

Ethical approval for this study was granted by the Tianjin First Central Hospital Medical Ethics Committee, and the trial was registered at the Chinese Clinical Trial Registry (www.chictr.org; ChiCTR1900025374, ‌25th August 2019, retrospectively registered).

### Treatment plan

**Fig. 1:**
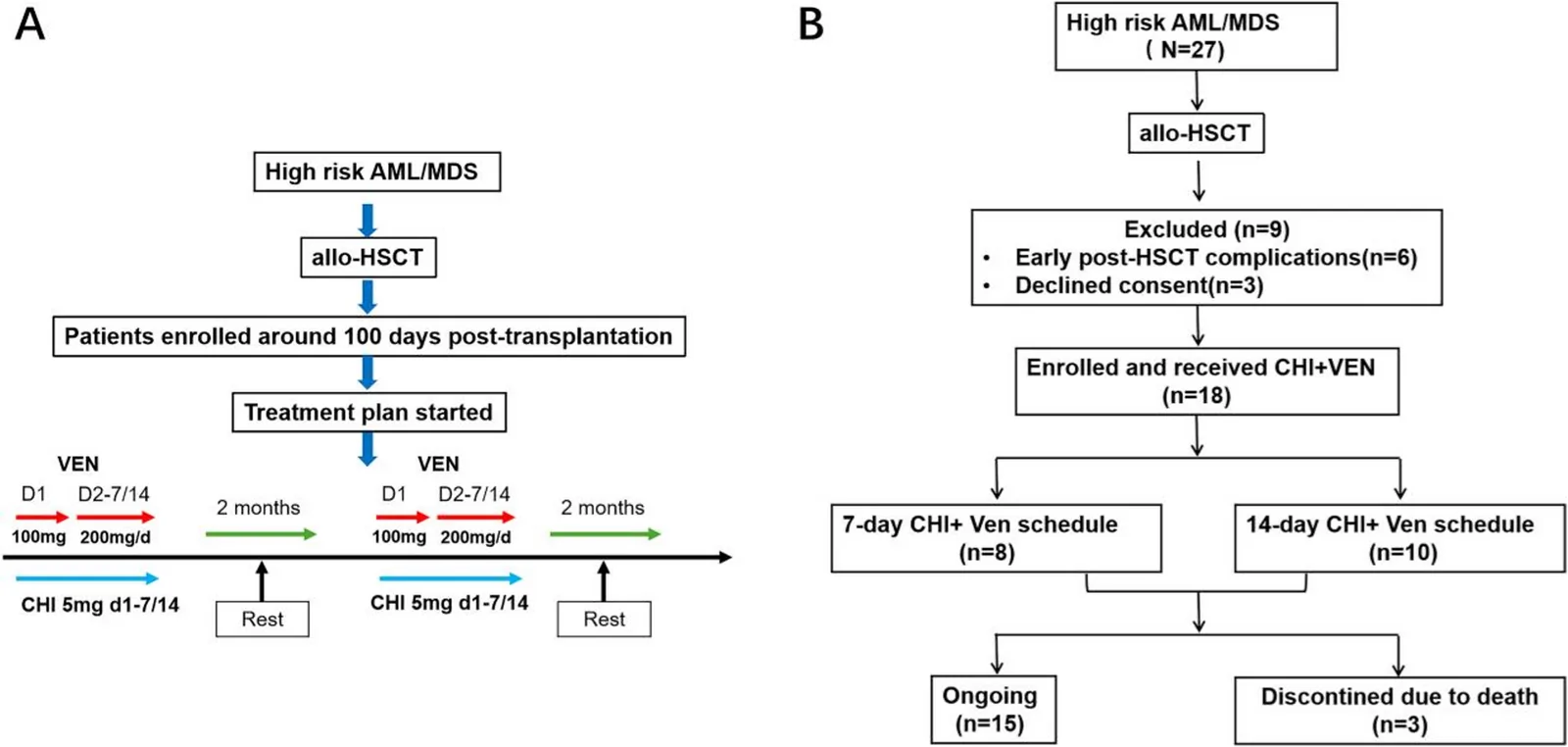
**A** Patient enrollment flow chart and treatment plan; **B** The CONSORT-style flowchart of the study

All patients included in this study underwent maintenance therapy combining CHI and VEN. The treatment schedule involved CHI at 5 mg/day from days 1 to 7 or days 1 to 14, along with VEN administered as a 100 mg dose on day 1, followed by 200 mg/day from days 2 to 7 or days 2 to 14, in combination with triazole antifungals. The 7 or 14 days choice was individualized based on each patient’s post-transplant hematologic recovery, bone marrow reserve, and overall performance status. Specifically, patients with adequate hematologic recovery, defined as an absolute neutrophil count ≥ 1.0 × 10⁹/L and platelet count ≥ 50 × 10⁹/L, and no active infections were preferentially assigned to the 14-day schedule to maximize potential efficacy. In contrast, patients with slower hematologic recovery, mild cytopenias, or concurrent mild infections received the 7-day schedule to ensure better tolerability and safety. Maintenance therapy was generally initiated around 100 days after allo-HSCT in high-risk AML/MDS patients. Treatment cycles were repeated at 2-month intervals until disease progression or unacceptable toxicity occurred (Fig. 1A). During treatment, patients were monitored for complete blood count, hepatic and renal function, drug concentrations, and other relevant indicators. Dosage adjustments were made based on laboratory results and patient tolerance. Treatment was promptly interrupted or discontinued in cases of intolerable adverse reactions. A CONSORT-style flowchart outlining patient screening, enrollment, treatment allocation, and reasons for discontinuation is presented in Fig. 1B.

### Efficacy and safety assessments

Efficacy evaluations during maintenance therapy were conducted before each treatment cycle and included the following assessments from the previous cycle: peripheral blood and bone marrow smears, immunophenotyping, gene mutations or fusion gene analysis, bone marrow chimerism (STR), and lumbar puncture. Long-term survival endpoints assessed were Overall Survival (OS), Disease-Free Survival (DFS), Cumulative Incidence of Relapse (CIR), and Non-Relapse Mortality (NRM). OS was defined as the time from the first dose of maintenance therapy to death from any cause or the last follow-up. DFS was defined as the duration from the initiation of maintenance therapy until the occurrence of MRD positivity or patient death. CIR represented the proportion of patients who experienced relapse or disease progression during the maintenance therapy period. NRM referred to the proportion of patients who died without prior relapse or disease progression during maintenance therapy.

In this study, all adverse events were graded and standardized in accordance with the Common Terminology Criteria for Adverse Events (CTCAE), version 5.0, published by the U.S. Department of Health and Human Services in 2017. All observed symptoms and signs of adverse reactions were meticulously assessed using this framework and documented according to its defined severity grades. These encompassed, but were not limited to, diarrhea, fatigue, rash, hematologic toxicity, hepatic insufficiency, renal insufficiency, cardiac insufficiency, and electrolyte disturbances. Additionally, the study evaluated the effect of maintenance therapy on the incidence of GVHD, as well as the rates of dose reduction, treatment delay, and therapy discontinuation attributable to the adverse reactions described above.

### Data collection

Patient demographic information was collected through the digital medical record management system at Tianjin First Central Hospital. This included, but was not limited to, gender, age, present illness history, past medical history (such as chronic conditions like hypertension, diabetes, and hyperlipidemia), and physical examination records. Laboratory test results, including complete blood count, biochemical indicators such as lactate dehydrogenase (LDH), aspartate aminotransferase (AST), alanine aminotransferase (ALT), total bilirubin (TBIL), blood urea nitrogen (BUN), as well as bone marrow cytology, were obtained via the blood test reporting software. Imaging results were retrieved by reviewing the hospital’s imaging and graphic report information system to assess disease progression and the status of tissues and organs. These included abdominal CT scans and their corresponding reports, color Doppler ultrasounds of the liver and spleen, cranial CT or MRI imaging, as well as detailed reports on bone marrow pathology and other related examinations. Follow-up survival data were obtained by reviewing patient medical records and/or conducting telephone interviews. The follow-up period concluded on March 1, 2025.

### Statistical analysis

In this study, data collection and initial management of raw data were performed using Microsoft Excel. Efficacy endpoints were assessed using Kaplan–Meier analysis, which provided estimates of survival rates at different time points, median survival time along with its 95% confidence interval, and corresponding survival curves. Continuous variables were summarized using descriptive statistics, including mean, standard deviation, median, and range (minimum to maximum), to characterize their distributions. Categorical data were presented as frequencies and percentages. To explore the relationships among study variables, COX regression analysis was conducted using SPSS software, and hazard ratios were estimated. Statistical significance was defined as follows: a P-value ≥ 0.05 indicated no statistically significant difference between groups; a P-value < 0.05 was considered statistically significant; and a P-value < 0.01 was deemed highly statistically significant.

## Results

### Cohort characteristics

A total of 18 patients had been enrolled in this study. Clinical data were collected to analyze baseline characteristics. The cohort consisted of 9 male and 9 female patients, with a median age of 40 years (range: 17–62 years). By disease classification, one patient was diagnosed with high-risk MDS, one had progressed from MDS to AML, and the remaining 16 had de novo AML. All patients were classified as having high-risk AML/MDS. Regarding high-risk features, there were 2 patients older than 60 years, 14 had r/rAML, 11 exhibited complex karyotypes, and 16 carried poor-prognosis gene mutations at initial diagnosis. Prior to transplantation, 13 were in complete remission (CR), 2 patients were in partial remission (PR), and the remaining 3 were in no response (NR), defined according to the ELN 2022 criteria [21]. PR was defined as all hematologic criteria of CR with decrease of bone marrow blast percentage to 5% to 25%, and decrease of pre-treatment bone marrow blast percentage by at least 50%. NR was defined as failure to meet the criteria for CR or PR. Among the 18 patients, 11 had achieved CR with MRD negativity, while 7 patients were MRD-positive. All patients received conditioning regimens according to the modified Beijing Protocol [22]. The conditioning regimen consisted of busulfan (Bu), cyclophosphamide (Cy), fludarabine (Flu), and cytarabine (Ara-C) in combination with anti-thymocyte globulin (ATG) for T-cell depletion. Graft-versus-host disease (GVHD) prophylaxis comprised cyclosporine A (CsA), mycophenolate mofetil (MMF), and short-term methotrexate (MTX). All patients received peripheral blood-derived CD34⁺ stem cells as the graft source (5 from HLA-matched siblings, 12 from haploidentical donors, and 1 from an unrelated donor). Anti-thymocyte globulin was administered for T-cell depletion prior to HSCT. The median number of infused CD34⁺ stem cells was 4.79 × 10⁶/kg (range: 2.25–9.60 × 10⁶/kg). The median time to neutrophil engraftment was 12 days (range: 10–15 days), and to platelet engraftment was 17 days (range: 14–23 days). See Table 1 for details.

**Table 1:** Cohort characteristics

Cohort characteristics	*N* = 18
Age [years, M (range)]	40(17–62)
Gender	
Male (n,%)	9(50.0)
Female (n,%)	9(50.0)
Disease type	
AML (n,%)	16(88.9)
MDS transformed to AML (n,%)	1(5.6)
MDS (n,%)	1(5.6)
Disease status at transplant	
NR(n,%)	3(16.7)
PR(n,%)	2(11.1)
CR(n,%)	13(72.2)
MRD-(n,%)	11(61.1)
MRD+(n,%)	7(38.9)
High-risk factors	
Refractory/relapsed (n,%)	14(77.8)
> 60 y old (n,%)	2(11.1)
Complex cytogenetic aberrations (n,%)	11(61.1)
poor-prognosis gene mutations at initial diagnosis(n,%)	16(88.9)
WBC > 100 × 10^9^/L at diagnosis (n,%)	2(11.1)
WPSS points ≥ 3 or IPSS-R points > 4.5(n,%)	1(5.6)
Median CD34^+^ cells at transplant [×10^6^, M (range)]	4.79(2.25—9.60)
Donor type	
Matched sibling donor (n,%)	5(27.8)
Haplo HLA-matched donor (n,%)	12(66.7)
Unrelated donor(n,%)	1(5.6)
Median days of neutrophils ≥ 0.5 × 10^9^/L after transplant [days, median (range)]	12(10–15)
Median days of platelets ≥ 20 × 10^9^/L after transplant [days, median (range)]	17(14–23)
Median follow-up time [days, range]	708(136–1254)

### Clinical outcomes

**Fig. 2:**
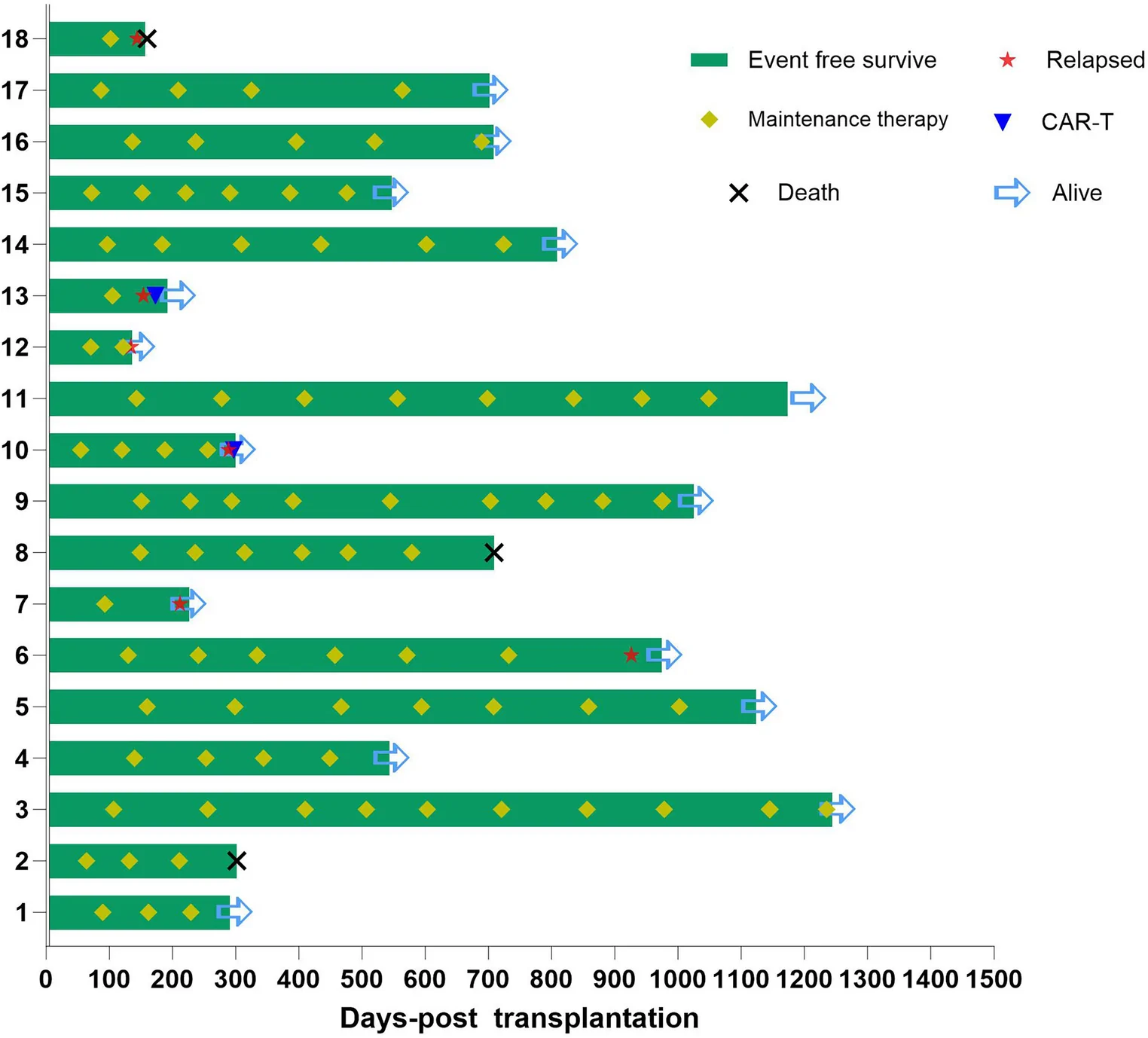
Clinical outcome of maintenance therapy after transplantation for 18 enrolled patients

All 18 subjects included in the study were scheduled to begin maintenance therapy with CHI in combination with VEN at approximately 100 days post-transplantation. The median time from transplantation to initiation of maintenance therapy was 112 days (range, 78–165 days). Patients received a median of 4 treatment cycles (range, 1–10). While all 18 patients received at least one cycle, only one patient received 10 cycles. Figure 2 summarizes the use of post-transplant CHI plus VEN as maintenance therapy and the outcomes among the 18 patients.

Among the 18 enrolled patients, a total of 86 cycles were administered, of which 57 cycles (66%) were delivered as scheduled without modification. Eight patients (44.4%) experienced delays in subsequent cycles due to grade 2–4 adverse events, including bacterial infection (*n* = 2), viral infection (*n* = 3), fungal infection (*n* = 1) and aGVHD (*n* = 2). Treatment delays, defined as an interval exceeding the planned 2-month cycle by more than 14 days, occurred in 8 patients across 29 cycles, with a median delay of 60 days (range, 30–120 days). Of note, in all 8 patients who experienced delays, the duration of VEN and CHI administration was reduced from 14 days to 7 days per cycle. No patient permanently discontinued treatment due to toxicity. Collectively, these data suggest that although deviations from the scheduled regimen occurred, they were predominantly driven by moderate infections and aGVHD, and were effectively managed with appropriate anti-infective and anti-GVHD therapy. Importantly, the 7-day schedule appeared to be better tolerated by patients.

As of March 2025, 10 patients remained in CR, 5 had experienced relapse, and 3 had died. Only one death was attributed to relapse of the primary disease; the other two deaths were due to severe infection and multiple organ failure.

Patient 2 maintained CR of the primary disease, MRD negativity, and high bone marrow chimerism throughout 3 cycles of maintenance therapy. Nevertheless, the patient died of respiratory failure resulting from a fatal episode of severe bacterial pneumonia. In the case of patient 10, 3 cycles of maintenance therapy was discontinued 200 days after transplantation. Subsequent follow-up assessments revealed recurrence of the *MLL/AF10* mutant gene. The patient was reinitiated on the fourth cycle of maintenance therapy; however, disease relapse occurred on day 289 post-transplantation. Salvage treatment with CLL1 CAR-T cell therapy was administered, and at the last follow-up, the patient remained in remission. For patient 18, bone marrow examination on day 102 post-transplantation indicated MRD-negative CR, though bone marrow chimerism was only 89.94%. The first cycle of maintenance therapy was administered as scheduled. However, before the second cycle began, relapse of the primary disease was detected. The patient presented with a high tumor burden, suggesting a very poor prognosis. Salvage therapy involving infusion of donor CD34⁺ stem cells was performed. Unfortunately, the patient died due to disease relapse on day 157 post-transplantation.

**Fig. 3:**
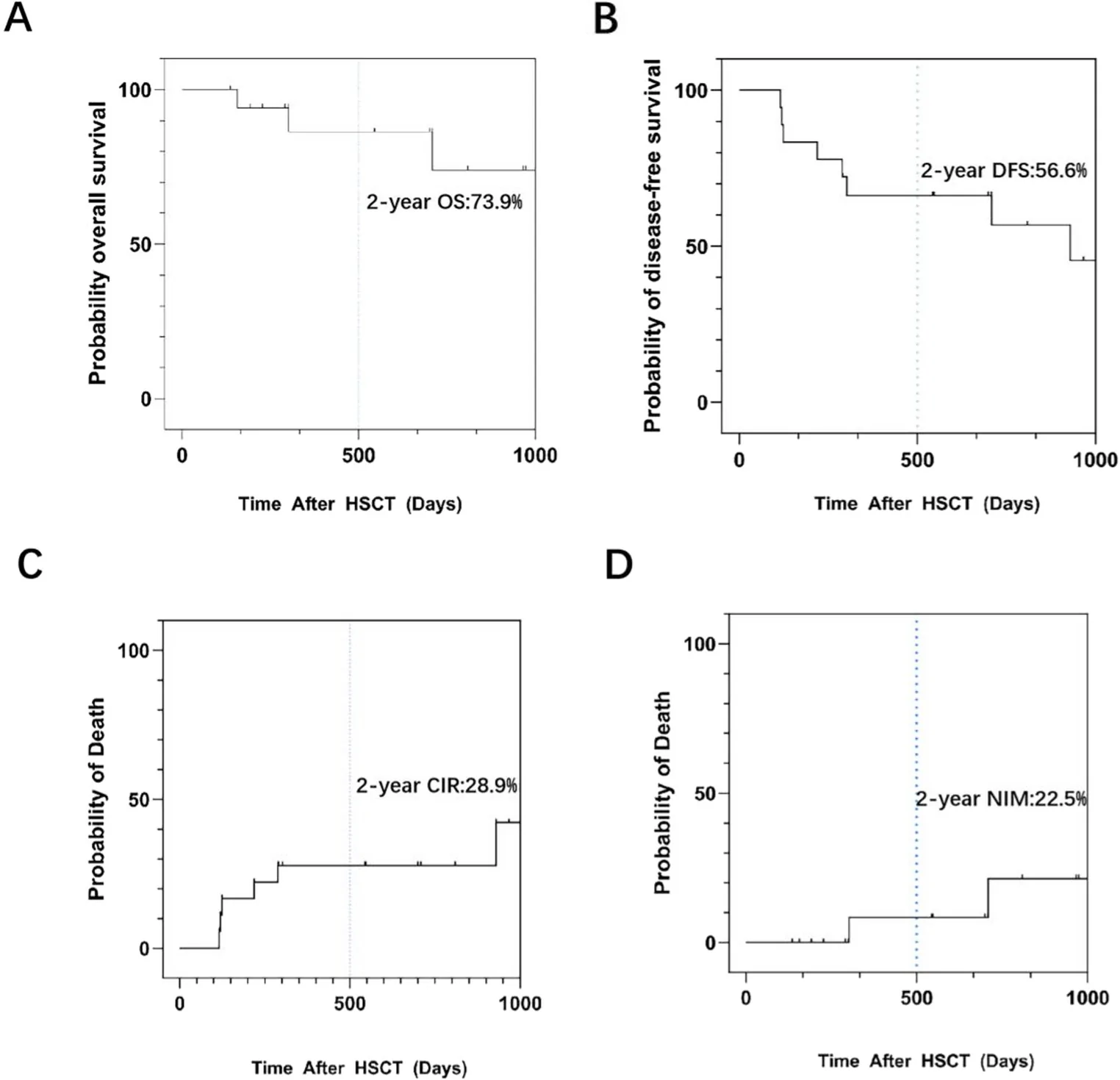
**A** and **B** 2-Year overall survival and disease-free survival; **C** and **D** 2-Year cumulative incidence of relapse and non-relapse mortality

The actual median follow-up time was 708 days post-transplantation (range, 136–1254 days). Among the high-risk AML/MDS patients in this study who received post-transplant maintenance therapy with CHI combined with VEN, the 2 year OS and DFS rates were 73.9% and 56.6%, respectively (Fig. 3A and B). The 2-year CIR was 28.9%, and the 2-year NRM rate was 22.5% (Fig. 3C and D).

### Drug-related adverse reactions

The common adverse reactions associated with CHI primarily include thrombocytopenia, neutropenia, anemia, gastrointestinal reactions (such as vomiting and diarrhea), and impaired hepatic and renal function. In this study, we systematically documented and assessed the occurrence of adverse events following each planned cycle of maintenance therapy. Results showed that hematological toxicities were the most frequently observed, aligning with previous reports on the principal toxicities of CHI. Among the 18 patients undergoing maintenance therapy, the main hematological adverse events included neutropenia (13 cases), anemia (7 cases), and thrombocytopenia (7 cases) (Table 2).The median duration of grade 3 to 4 neutropenia was 9 days, ranging from 5 to 18 days, and the median duration of grade 3 to 4 thrombocytopenia was 7 days, ranging from 3 to 14 days. Additionally, several non-hematological adverse reactions were recorded, such as fatigue (6 cases), diarrhea (11 cases), and neutropenia-associated fever (5 cases), as summarized in Table 2. Regarding organ toxicities, 3 cases of hepatic impairment (2 grade 1 and 1 grade 2), 3 cases of renal impairment (2 grade 1 and 1 grade 2), and 2 cases of cardiac toxicity (1 grade 2 and 1 grade 3) were observed. Importantly, no grade 4 or grade 5 cardiac, renal, or hepatic toxicities occurred in this study. In addition, no treatment related deaths were reported, and no instances of tumor lysis syndrome (TLS) were observed among the patients. No patients discontinued treatment due to toxicity, which further supports the manageable safety profile of this regimen.

**Table 2:** Adverse events during maintenance therapy with chidamide plus venetoclax

System Organ Class / Adverse Event	Grade 1–2	Grade 3	Grade 4	Grade 5	Total
Hematologic disorders					
Anemia	4	3	0	0	7
Thrombocytopenia	4	2	1	0	7
Neutropenia	6	5	2	0	13
Febrile neutropenia	3	2	0	0	5
Cytopenia (overall)	14	10	3	0	27
Gastrointestinal disorders					
Nausea/Vomiting	3	2	0	0	5
Diarrhea	6	3	2	0	11
Stomatitis/Mucositis	2	1	0	0	3
Colitis/Enteritis	1	1	0	0	2
Hepatobiliary disorders					
Hepatic insufficiency/ Liver enzyme elevation	2	1	0	0	3
Renal disorders					
Renal insufficiency/ Creatinine elevation	2	1	0	0	3
Cardiac disorders					
Cardiac insufficiency/Arrhythmia	1	1	0	0	2
Infections					
Bacterial infection	3	2	0	0	5
Viral infection	4	3	1	0	8
Fungal infection	0	1	0	0	1
Respiratory disorders					
Pneumonia (all causes)	2	2	1	1†	6
Respiratory failure	0	0	0	1†	1
Constitutional / Other					
Fatigue/Asthenia	3	2	1	0	6
Electrolyte imbalance	7	2	0	0	9
Multi-organ failure	0	0	0	1	1
Hospitalization events	0	1	4	3	8

† The deaths were not related to the maintenance therapy

### GVHD

For GVHD prophylaxis, cyclosporine A, methotrexate, and mycophenolate mofetil were administered. For the treatment of GVHD, most patients received a combination of cyclosporine A and oral corticosteroids, with doses of both agents adjusted according to the individual’s clinical status. In some cases, tacrolimus was used as an alternative to cyclosporine A. Given the current lack of studies on the CHI + VEN regimen in the context of GVHD, we also documented the incidence of GVHD before and after the maintenance therapy. 6 patients suffered from acute GVHD (aGVHD) before our treatment plan started, 2 patients had aGVHD after the maintenance therapy, with grade 2 and 3 being the most common (Table 3). Chronic GVHD (cGVHD) was observed in three patients, affecting the skin, the small joints of the hands, and the oral cavity, respectively, which were mild and limited according to the National Institutes of Health (NIH) classification. All cases were alleviated following anti-rejection therapy. These findings suggest that the use of CHI combined with VEN for post-transplant relapse prevention did not lead to severe GVHD.

**Table 3:** aGVHD before and after the maintenance therapy

aGVHD before the maintence therapy	Grade 1	Grade 2	Grade 3	Grade 4
Total	1	2	3	0
Skin	0	1	2	0
Gastrointestinal tract	1	0	1	0
Liver	0	1	0	0
aGVHD after the maintence therapy	Grade 1	Grade 2	Grade 3	Grade 4
Total	0	2	0	0
Skin	0	1	0	0
Gastrointestinal tract	0	1	0	0
Liver	0	0	0	0

### Infection complications

In this study, a total of 3 patients died, with infection being the cause of death in 2 cases. During the maintenance treatment period, 5 patients developed at least one bacterial infection, and 1 patient had a fungal infection (mucormycosis), with incidence rates of 28% and 5%, respectively. There were 6 cases of CMV viremia, including 2 patients with a history of CMV infection prior to maintenance therapy and 1 who developed CMV pneumonitis, along with 1 case of EBV viremia, yielding incidence rates of 33% for CMV and 5% for EBV infection, respectively. All patients with CMV or EBV viremia received following antiviral therapy or lymphocyte depleting therapy, and viruses turned negative finally. One patient contracted COVID-19 and recovered after antiviral treatment.

## Discussion

Disease relapse remains the primary factor impairing survival outcomes in high-risk AML/MDS patients after HSCT. It not only leads to low re-remission rates but also diminishes long-term survival, contributing to poor prognoses [23–27]. To address this challenge, numerous studies have proposed maintenance therapy during the post-transplant immune reconstitution phase as a strategy to prevent relapse and improve patient outcomes [3, 25, 28]. However, no consensus exists on the optimal maintenance regimen. Established approaches are increasingly limited by drug resistance and treatment failure, while novel agents remain inadequately studied in terms of dosing, timing, and toxicity profiles [3, 25, 28]. In this study, we introduced a novel maintenance therapy combining CHI and VEN in 18 high-risk AML/MDS patients. Preliminary results suggest that this regimen did not induce severe toxicities or significantly increase the incidence of GVHD. Furthermore, it was associated with reduced rates of relapse and sustained long-term remission.

The Ven-HMAs combination is currently approved for newly diagnosed AML patients ineligible for intensive chemotherapy and is increasingly being explored across various stages of AML treatment [25, 26]. In recent years, this combination has attracted growing interest as a maintenance therapy for high-risk AML/MDS patients following allo-HSCT, owing to its dual mechanism of synergistically inhibiting the anti-apoptotic protein BCL-2 and reversing DNA hypermethylation [8, 28, 29]. Over the past three years, our center has pioneered the use of the BCL-2 inhibitor VEN combined with hypomethylating agents (decitabine or azacitidine) as maintenance therapy to prevent relapse in high-risk AML/MDS patients after allo-HSCT, yielding benefits for the majority of transplant recipients [30].

Despite these advances, clinical experience has revealed that some patients undergoing Ven-HMAs maintenance still experience disease progression or develop resistant relapse, accompanied by short median survival and high mortality [9, 11]. Such treatment failures are often linked to high-risk genetic features, including TP53 mutations and specific fusion genes [10–12, 31]. This is particularly evident in acute monocytic leukemia, where patients generally exhibit poor sensitivity to BCL-2 inhibitors [10–12, 31]. A retrospective analysis further indicated that monocytic AML patients with genetic abnormalities such as TP53 mutations or AML1-ETO positivity are more prone to developing resistance to VEN [32]. In response to Ven-HMAs resistance, a prior report documented that the addition of the histone deacetylase inhibitor CHI could reverse resistance and restore treatment sensitivity in three patients who had failed Ven-HMAs therapy, leading to renewed remission [33]. At our center, we have expanded upon these findings with a larger sample size, reinforcing the evidence that CHI serves as an effective salvage therapy following Ven-HMAs failure (data not shown). Moreover, a prospective study (*n* = 48) presented at the 2024 American Society of Hematology meeting showed that the CVA regimen (CHI + VEN+azacitidine) significantly improved the complete remission rate compared to the VA regimen (VEN+azacitidine) in newly diagnosed AML patients unsuitable for intensive chemotherapy, despite an increase in grade 3 hematological toxicity [34].

Building on this foundation, we have proposed a combined CHI and VEN strategy and demonstrated its potential as a novel maintenance therapy to prevent relapse in high-risk AML/MDS patients post-transplantation. The CHI-VEN combination represents a multidimensional innovation in overcoming BCL-2 inhibitor resistance and improving treatment outcomes for AML [35]. As an oral histone deacetylase inhibitor, CHI enhances VEN-induced apoptosis through epigenetic remodeling [36, 37]. This regimen acts synergistically to overcome drug resistance through multiple mechanisms, including: (1) co-targeting key signaling pathways such as PI3K/AKT and JAK2/STAT3, thereby cooperatively promoting apoptosis in AML cell lines and primary cells [14, 19]; (2) promoting DNA double-strand breaks and modulating the balance between anti-apoptotic and pro-apoptotic proteins, for instance by downregulating MCL-1 and Bcl-xL while upregulating Bim to synergize with VEN [20]; and (3) inhibiting the expression of key glycolytic enzymes to deplete energy in AML cells and further enhance treatment efficacy [19, 37].

In conventional VEN+ azacitidine maintenance therapy, treatment is administered on a monthly cycle with a fixed-duration approach [38], whereas our CHI + VEN maintenance regimen includes a 2-month treatment-free interval until disease progression or unacceptable toxicity occurs. The 2-month interval between cycles was adopted based on several considerations. First, given the potential for synergistic myelosuppression with the CHI and VEN combination, extending the treatment free interval to 2 months allows adequate time for hematologic recovery and reduces the risk of cumulative bone marrow toxicity. This is particularly important in the post-transplant setting, where marrow reserve may be limited. Second, as an epigenetic modulator, CHI may exert sustained biological effects beyond the active dosing period [39], supporting an intermittent schedule without compromising therapeutic efficacy. Third, a less frequent dosing schedule improves patient convenience and adherence, which is critical for long term maintenance therapy. Thus, while the standard VEN plus HMA regimen is administered monthly, we opted for a 2-month interval to balance efficacy, safety, and quality of life in this high-risk transplant population.

Several limitations of this study should be acknowledged. First, this was a retrospective, single arm analysis with a small sample size of only 18 patients, which inherently limits statistical power and generalizability. The modest enrollment over the 4-year study period reflects the highly selective nature of our cohort, as we focused exclusively on high-risk AML/MDS patients who had successfully undergone allo-HSCT and were suitable for maintenance therapy. We applied rigorous exclusion criteria to rule out patients with early post-transplant complications such as grade III to IV aGVHD, severe infections, or engraftment failure within 100 days. Additionally, some otherwise eligible patients declined therapy due to financial or personal reasons. Consequently, a selection bias is likely present, meaning our enrolled patients may represent a relatively healthier subgroup with favorable post-transplant recovery and fewer comorbidities, all of which could have positively influenced the observed outcomes. Second and equally important, the absence of a concurrent or historical control arm precludes any direct comparison with alternative maintenance strategies such as azacitidine-VEN or HMA monotherapy. We therefore do not claim superiority of the CHI-VEN regimen over any existing approach. Instead, our findings should be interpreted as preliminary evidence of clinical activity and acceptable safety, providing a strong rationale for designing future randomized controlled trials or well-matched prospective cohort studies to directly compare the CHI-VEN regimen with current standard of care maintenance options.

## Conclusion

The combination of CHI and VEN may represent a novel strategy to overcome drug resistance in high-risk AML/MDS through epigenetic and metabolic modulation. As post-transplant maintenance therapy, this regimen has demonstrated encouraging signals of efficacy in terms of overall survival and relapse reduction, with a manageable safety profile in our cohort. However, given the exploratory nature and limited sample size of this study, these findings should be interpreted with caution. Larger prospective studies with extended follow-up are needed to validate its long-term efficacy and safety.

## Data Availability

No datasets were generated or analysed during the current study.
